# Spinal cord stimulation in the treatment of refractory angina: systematic review and meta-analysis of randomised controlled trials

**DOI:** 10.1186/1471-2261-9-13

**Published:** 2009-03-25

**Authors:** Rod S Taylor, Jessica De Vries, Eric Buchser, Mike JL DeJongste

**Affiliations:** 1 Health Services Research, Peninsula Medical School, Universities of Exeter & Plymouth, Exeter, UK; 2 Department of Cardiology, Thorax Centre, University Medical Centre Groningen, The Netherlands; 3 Pain Clinic, Morges Hospital, Morges, Switzerland

## Abstract

**Background:**

The aim of this paper was undertake a systematic review and meta-analysis of the use of spinal cord stimulation (SCS) in the management of refractory angina.

**Methods:**

We searched a number of electronic databases including Medline, Embase and Cochrane Library up to February 2008 to identify randomised controlled trials (RCTs) reporting exercise capacity, ischemic burden, functional class, quality of life, usage of anti-anginal medication, costs and adverse events including mortality. Results were reported both descriptively for each study and using random effects meta-analysis. Given the variety in outcomes reported, some outcome results were pooled as standardised mean differences (SMD) and reported in standard deviation units.

**Results:**

Seven RCTs were identified in a total of 270 refractory angina patients. The outcomes of SCS were found to be similar when directly compared to coronary artery bypass grafting (CABG) and percutaneous myocardial laser revascularisation (PMR). Compared to a 'no stimulation' control, there was some evidence of improvement in all outcomes following SCS implantation with significant gains observed in pooled exercise capacity (SMD: 0.76, 0.07 to 1.46, *p *= 0.03) and health-related quality of life (SMD: 0.83, 95% CI: 0.32 to 1.34, *p *= 0.001). Trials were small and were judged to range considerably in their quality. The healthcare costs of SCS appeared to be lower than CABG at 2-years follow up.

**Conclusion:**

SCS appears to be an effective and safe treatment option in the management of refractory angina patients and of similar efficacy and safety to PMR, a potential alternative treatment. Further high quality RCT and cost effectiveness evidence is needed before SCS can be accepted as a routine treatment for refractory angina.

## Background

The term 'refractory angina' is defined as "a chronic condition caused by clinically established reversible myocardial ischemia in the presence of coronary artery disease, which cannot be adequately controlled by a combination of medical therapy, angioplasty or coronary artery bypass operations" [1]. Both increasing success and innovation in conventional approaches to treat angina and better survival rates following primary and subsequent coronary events have led to significant proportions of patients presenting with angina refractory to conventional treatment. It is estimated that in Europe the incidence of refractory angina is 100.000 new cases per year [2].

For this patient group, a number of non-conventional treatment options have emerged including, pharmacotherapy, enhanced external counterpulsation, percutaneous myocardial laser revascularisation (PMR), percutaneous coronary artery bypass (CABG), and spinal cord stimulation (SCS) [1,3].

First described for angina in 1987, SCS, is a reversible procedure in which electrodes are implanted in the epidural space to stimulate the dorsal columns of the spinal cord [4]. The technique has been described in detail elsewhere [4,5]. SCS has been successfully used to relieve pain in a number of chronic conditions including neuropathic pain and peripheral vascular disease [5-7].

Published reviews have suggested the clinical efficacy of SCS in refractory angina [8-12]. SCS has been shown to provide chronic refractory angina patients with symptomatic relief that is equivalent to CABG, with lower rates of complications and re-hospitalisation. Despite these conclusions and a level B (evidence class IIb) recommendation in 2002 American College of Cardiology; American Heart Association (ACC/AHA) guidelines concerning chronic stable angina [13] and a level A recommendation by European Society of Cardiology (ESC) Joint Study Group on the treatment of refractory angina [1], the technique has had little adoption by the cardiology community [10]. One reason may be the absence to date of an authorative review incorporating a meta-analysis of existing evidence.

The aim of this study was to undertake systematic review and meta-analysis of randomised controlled trials (RCTs) to assess the efficacy, safety and cost effectiveness of SCS in patients with refractory angina.

## Methods

The review was undertaken in accord with the methods of The Cochrane Collaboration [14].

### Search strategy

The following electronic databases were searched: MEDLINE (Ovid) 1950 – January week 5 2008, MEDLINE In Process and other Non-Indexed citations (Ovid) at February 08 2008, EMBASE (Ovid) 1980 – 2008 week 6, Cochrane Library (Wiley) 2008 Issue 1 (CDSR, DARE, CENTRAL, NHS EED, HTA databases). The metaRegister (Current Controlled Trials) and ClinicalTrials.gov were searched to identify ongoing and unpublished research. Internet sites of national and international health technology assessment organisations were also searched. We sought unpublished literature by searching the Internet sites of regulating authorities (Food and Drug Administration, and European Medicines Evaluation Agency) and by contacting experts in the field.

The search strategy was developed to maximise the sensitivity of article identification. We incorporated both controlled vocabulary (e.g., Medical Subject Headings [MeSH]) and key words ('refractory angina pectoris and [synonym]' and 'spinal cord stimulation or neuromodulation or [synonym]'). An example search strategy is shown in Appendix 1.

### Inclusion criteria and data extraction

Studies were considered eligible if they were RCTs (parallel or cross over); included patients with refractory angina (according to pre-defined clinical criteria) and received SCS (either alone or in combination with other therapies). Three categories of outcome were sought i.e. efficacy – exercise capacity, ischemic burden (combined frequency and severity of ischemic changes during ambulatory ECG monitoring), anti-anginal drug consumption, functional class and health-related quality of life; safety – mortality, morbidity and SCS-related complications; and healthcare utilisation or costs. No language restrictions were applied in the selection of studies. Non-randomised studies were excluded as were studies that reported only physiological/experimental outcomes (e.g. myocardial blood flow) or studies using transcutaneous electrical nerve stimulation.

Two reviewers undertook study selection and data extraction using a standardised data proforma. Where multiple time points were reported, all follow up data were considered. Study authors were contacted to seek clarification on issues of reporting or to provide further outcome details.

### Risk of bias assessment

Each included trial was evaluated with regard to whether there was an appropriate method of randomisation (e.g., statement of computer-generated numbers) and an adequate concealment of randomisation (e.g., randomisation codes been kept from those involved in running the trial), whether there was blinding (particularly blinding of outcome given the difficulties of blinding patients as the result of parasthesia), whether the losses to follow-up/drop-outs were reported and if so, whether this rate was < 20%, and whether an intention-to-treat (ITT) analysis was performed [14]. If a trial did not report these parameters, then the criteria were judged as unmet (i.e., 'not reported', NR). In additional to a qualitative assessment, a total risk of bias score was obtained using a modified version of the Jadad scale [15]. Risk of bias was assessed by two independent reviewers.

### Data presentation and analysis

Findings of this review are presented according to the two broad categories of control group i.e. SCS versus active intervention (i.e. CABG or PMR) and active SCS ('SCS ON') versus no or inactive SCS ('SCS OFF'). A two stage approach to data synthesis was taken. First the findings of all the studies were summarised qualitatively according to their direction of effect and statistical significance of both within group differences (i.e. comparison of outcome at baseline and follow up in the SCS and control groups) and between group difference (i.e. comparison of outcomes in SCS group versus control group). If the within group change was not reported in the source paper, it was calculated making allowance for the within-patient correlation from baseline to follow-up measurements [14,16]. Results were only used from the first period of cross-over trials [14].

The between group differences of active SCS ('SCS ON') versus no or inactive SCS ('SCS OFF') trials were therefore quantitatively pooled using meta-analysis. A conservative Der Simonian random effects meta-analysis model was used to take account of the potential heterogeneity (both clinical and methodological) across trials [17]. Given the variety of outcome measures reported, results were expressed as a standardised mean difference (SMD). SMD is a summary statistic used when trials assess the same outcome, but measure it in a variety of ways (for example, all trials measure quality of life but they use different scales) [14]. The SMD expresses the size of the treatment effect in each trial relative to the study variance or standard deviation observed in the trial. To prevent the risk of carry over effects, only the first period of cross-over trials were included and the variance modified to take account of the paired nature of the between group comparison [14]. Funnel plots (i.e. scatter plots of the mean intervention effect versus the inverse of variance of the intervention effect for each study) was used to explore the possibility of publication bias [18]. All analyses were performed using RevMan, version 5.0 .

## Results

### Identification and selection of studies

**Figure 1:**
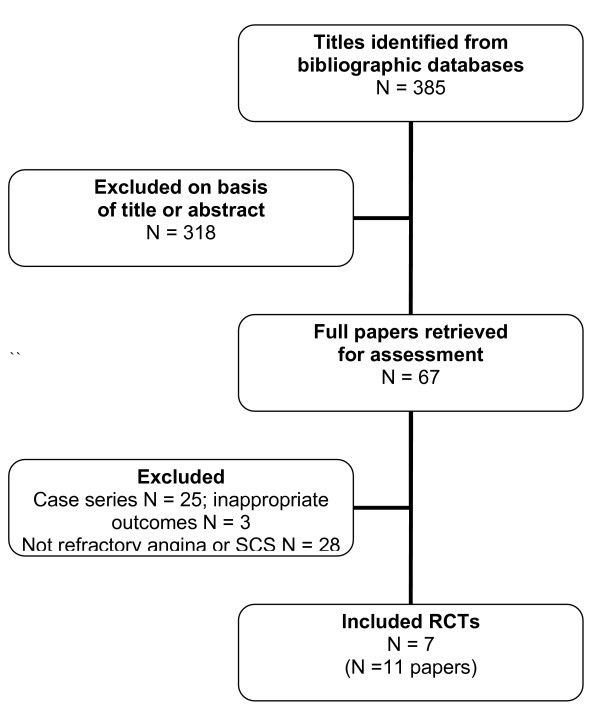
**Summary of study selection and exclusion process**.

Our bibliographic search yielded 385 titles. After reviewing these titles and abstracts, 67 full papers were retrieved for possible inclusion. Most abstracts and titles or full papers were excluded on the basis of an inappropriate intervention or that they were case reports. Eleven publications across seven RCTs (a total of 270 patients) were judged to meet the inclusion criteria (see Figure 1) [19-29]. The RCT of Lanza et al was excluded on grounds that it was based on patients with Syndrome X [30].

### Description of randomised controlled trials

Two studies compared SCS to an alternative active intervention: CABG – ESBY trial [19-22], and PMR – SPiRiT trial [23]. Four studies compared active SCS ('SCS ON') to inactive SCS ('SCS OFF') (DiPede et al, 2001 [26]; Hauvast et al, 1998 [27]; Jessurun et al, 1999 [28], Eddicks et al, 2007 [29]) and one study compared SCS to a control group receiving no SCS implantation (DeJongste et al, 1993 [[24], 35]) (see Table 1). Eddicks et al randomised patients into three SCS stimulation groups (i.e. 3 × 2 hours/day, 24 hours/day with conventional output, 3 × 2 hours/day with a subthreshold output) and control (24 hours/day with 0.1 volts) [29]. For the purposes of this review, the results from 3 × 2 hours/day group were used. Three studies were conducted in the same centre. One of the authors of this review (MdeJ) was an investigator in these studies and confirmed that there was no overlap in patents.

**Table 1:** Summary of included RCT characteristics

**Trial Name/author** Year, country Setting Design Recruitment dates	**Population** Age – Mean (SD or range) Sex – % male Class Inclusion criteria	**Intervention** N patients	**Control** N patients	**Relevant outcomes**	Follow up*
**de Jongste et al^24,25^** 1994, Netherlands Single Centre Parallel RCT Jan 1990 to March 1992	60 years (5) 71% male NYHA III/IV Angiographically confirmed CAD not suitable for revascularisation. Reversible ischemia on exercise. Receiving optimal drug therapy.	SCS N = 12	No SCS N = 10	Primary outcome: exercise capacity (treadmill time). Secondary outcomes: health related quality of life, nitrate drug usage, ischemic burden & adverse events	2-months
**Di Pede et al^26^** 2001, Italy Single Centre Cross over RCT Oct 1990 to Sept 1998	76 yrs (8) 60% male CCS 3/4 Severe angina despite optimal drug treatment. Revascularisation not possible. Reversible ischemia on exercise.	SCS ON N = 15	SCS OFF N = 15	Ischemic burden	48-hours
**ESBY^19–22^** 1998, Sweden Single centre Parallel group RCT Oct 1990 to Sept 1998	68.9 yrs (40–82) 80% male AHA angina class 3/4 Symptomatic indication for CABG & no prognostic benefit from CABG	SCS N = 53	CABG N = 51	Primary outcomes: exercise capacity (workload), angina attacks Secondary outcomes: nitrate drug usage, ischemic burden, health-related quality of life, morbidity, mortality & complications	6-months, 2-years, 5-years
**Hautvast et al^27^** 1998, Netherlands Single Centre Parallel group RCT Not reported	62.5 yrs (7.5 years) 56% male NYHA class III/IV Angiographically confirmed CAD not suitable for revascularisation with proven ischemia. Reversible ischemia on exercise. Receiving optimal drug therapy.	SCS ON N = 13	SCS OFF N = 12	Exercise capacity (treadmill time), angina attacks, nitrate drug usage & health-related quality of life	1.5 months
**Jessurun et al^28^** 1999, Netherlands Single Centre Parallel group RCT Not reported	59 yrs (5.5) 67% male NYHA class III/IV Unresponsive to optimal medication. Revascularisation not possible. Reversible ischemia on exercise or equivalent	SCS ON N = 12	SCS OFF N = 12	Exercise capacity (treadmill V0_2_max), ischemic burden, & nitrate drug usage	1-month
**SPiRiT** 2006, UK Single centre Parallel group RCT Dec 2000–Dec 2003	63.5 yrs (8) 88% male CCS class 3/4 Limiting angina despite optimal drug therapy. Angiography confirmed CAD and reversible ischemia on radionuclide scanning	SCS N = 34	Percutaneous myocardial laser revascularisation (PMR) N = 34	Primary outcome: exercise capacity (treadmill time). Secondary outcomes: functional class, mortality, quality of life, morbidity, mortality & complications	3-months, 12 months
**Eddicks et al^29^** 2007, Germany Single centre Cross over RCT June 2003 to August 2004	65 yrs (8) 67% male CCS 3/4 Proven responders to SCS, angina > 3 months, known CAD, reversible myocardial ischemia, receiving optimal drug therapy with no benefit from revascularisation	SCS ON^1^ N = 12	SCS OFF^2^ N = 12	Primary outcome: exercise capacity (6-min walk test) Secondary outcomes: number of angina attacks, health-related quality of life (Seattle Angina Questionnaire), CCS class, nitrate drug usage, drop out due to intolerable symptoms	4 weeks

*: follow up related to randomised comparison; CCS: Canadian Cardiovascular Scale; AHA: American Heart Association; CABG: coronary artery bypass graft.

1. consisted of 3 SCS states – stimulation for 3 × 2 h/day with conventional output, 24 h/day with conventional output, 3 × 2 h/day with subthreshold output

2. stimulation for 24 h/day with 0.1 V output

All studies were conducted in European centres and were single centre. Patient inclusion criteria appeared similar across studies i.e. all had experienced refractory angina in spite of optimal medical therapy and unable to undergo revascularisation, and angina class NHYA class III and IV or Canadian Cardiovascular Society (CCS) class 3 and 4. Included patients were predominantly male (63%) with a median age of 63.5 years, the majority of whom had experienced a previous myocardial infarction (median 73%). The majority of trials were of short term follow up (i.e. ≤ 2 months), the SPiRiT and ESBY studies reporting follow up of 1 year or longer.

### Risk of bias

The limited level of reporting method detail by many included studies hampered the full assessment of their risk of bias (see Table 2). Only one trial provided details of random sequence generation and two trials reported the method of allocation concealment. Although four studies employed a 'SCS ON' vs. 'SCS OFF' cross over design blinding is likely to have been prevented by patient's perception of paraesthesia. One study achieved double blinding by employing a very low stimulus control group (0.1 v 24 hours per day) [29]. One study reported blinded ECG outcome assessment [26]. There was little evidence of attrition bias, all studies reporting follow up on 90% or more of patients randomised. The median Jadad score was 2 (range, 2 to 4, out of a maximum score of 5) indicating overall, a moderate level of risk of bias.

**Table 2:** Risk of bias and funding source

	**Random sequence generation**	**Concealment of randomisation**	**Blinding**	**Loss to follow up/drop out**	**Method of data analysis**	**Jadad score (/5)**	**Funding**
**DeJongste 1994**	Not stated	Independent telephone service	Open label	8%	Not stated	2	Government
**DiPede 2001**	Not stated	Not stated	Blinded ECG assessment	0%	Not stated	3	Not stated
**ESBY 1998**	Not stated	Not stated	Open label	7%*	Intention to treat	2	Government
**Hauvast 1998**	Not stated	Not stated	Open label	0%	Not stated	2	Government
**Jessurum 1999**	Not stated	Not stated	Open label	0%	Not stated	2	Industry
**SPiRiT 2006**	Computer-generated	Independent R&D department	Open label	9%	Intention to treat	4	Industry
**Eddicks 2007**	Not stated	Not stated	Double blind	0%	Not stated	4	Industry

*: at 6-months follow up

### Outcomes

#### Exercise capacity

**Figure 2:**
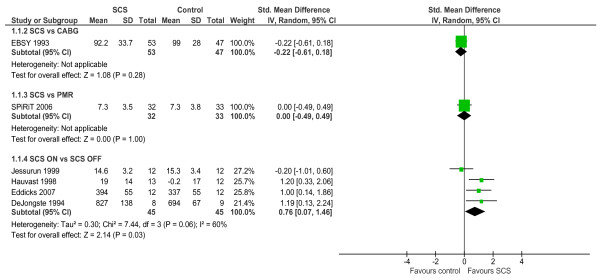
**Exercise capacity – between-group difference**.

Six studies assessed exercise capacity, reporting outcomes using a number of metrics (e.g. exercise duration, maximum oxygen uptake and peak workload) [19,23,24,27-29]. Of the four studies that reported the within group difference in outcome between baseline and follow up with SCS, three found a statistically significant improvement in exercise capacity (Table 3). Although in the ESBY study no significant change in maximum work capacity was observed 6-months post-implant, SCS was inactivated during the exercise test. As shown in Figure 2, in the pooled analysis there was a superior exercise capacity with active SCS compared to no SCS or SCS OFF (SMD: 0.76, 0.07 to 1.46, p = 0.03, heterogeneity test: χ^2 ^= 7.4, p = 0.06, I^2 ^= 60%). Compared to both CABG and PMR no significant difference was seen in exercise capacity scores at follow up. However, when adjusted for baseline values, the ESBY trial authors did report a small difference in exercise capacity at follow up in favour of CABG (p = 0.03).

**Table 3:** Qualitative summary of efficacy outcomes

**A. SCS vs. active comparator studies**				
**Trial Name/author**	**Outcome**	**Within group difference SCS**	**Within group difference Control**	**Between group difference SCS vs. control**

**ESBY 1998 (vs CABG)**	Nitrate drug usage	6-mo +	6-mo +	6-mo =
		2-yr +	2-yr +	2-yr =
	Exercise capacity	6-mo =	6-mo +	6-mo --
	Total mortality	NR	NR	6-mo +
		NR	NR	2-yr =
		NR	NR	5-yr =
	Non fatal morbidity	NR	NR	6-mo =
	Ischemic burden	6-mo =	6-mo =	6-mo =
	HRQoL (QLQ-AP & NHP)	6-mo +	6-mo +	6-mo =
		2-yr +	2-yr +	2-yr =
		6-mo +	6-mo+	6-mo =
	Angina			
SpiRiT 2006* (vs PMR)	Exercise capacity	3-mo +	3-mo =	3-mo =
		12-mo =	12-mo =	12-mo =
	CCS class	3-mo +	3-mo +	3-mo +
		12-mo +	12-mo +	12-mo =
	HRQoL (SF-36 &	3-mo +	3-mo +	3-mo =
	SAQ)*	12-mo +	12-mo +	12-mo =

**B. SCS vs no SCS/SCS OFF studies**				

**Trial Name/author**	**Outcome**	**Within group difference SCS**	**Within group difference Control**	**Between group difference SCS vs. control**

**DeJongste 1994**	Exercise capacity	+	=	+
	Nitrate drug usage	+	=	+
	HRQoL (ADL score)	+	=	+
**Hautvast 1998**	Exercise capacity	+	=	+
	Nitrate drug usage	+	=	=
	HRQoL (LASA)	+	=	=
	Ischemic burden	+	=	=
**Jessurun 1999**	Exercise capacity	NR	NR	=
	Ischemic burden	NR	NR	=
	Nitrate drug usage	NR	NR	=
**DiPede 2001 Eddicks 2007**	Ischemic burden	NR	NR	=
	Exercise capacity	NR	NR	+
	Nitrate drug usage	NR	NR	+
	HRQoL (SAQ)	NR	NR	+/=
	(EQ-5D)	NR	NR	+
	CCS class	NR	NR	+
	Angina attacks	NR	NR	+

*additional data supplied by authors

HRQOL: health-related quality of life; ADL: activities of daily living score; SF-35: Short-form 36 questionnaire; NHP: Nottingham Health Profile; QLQ-AP: Quality of Life Questionnaire – Angina Pectoris; SAQ: Seattle Angina Questionnaire; SAS: Social Activity Scale; LASA: Linear Analogue Self Assessment; VAS – visual analogue scale; 6-MWT: 6 minute walk test; NR: not reported

+: *within group *– improvement (P ≤ 0.05) in outcome with SCS or control at follow up compared to baseline; *between group *– superior (P ≤ 0.05) outcome at follow up with SCS compared to control

-: *within group *– worsening (P ≤ 0.05) in outcome with SCS or control at follow up compared to baseline; *between group *– superior (P ≤ 0.05) outcome at follow up with control compared to SCS

=: *within group *– no change (P > 0.05) in outcome with SCS or control at follow up compared to baseline; *between group *– no difference (P > 0.05) outcome at follow up of SCS compared to control

#### Ischemic burden

**Figure 3:**
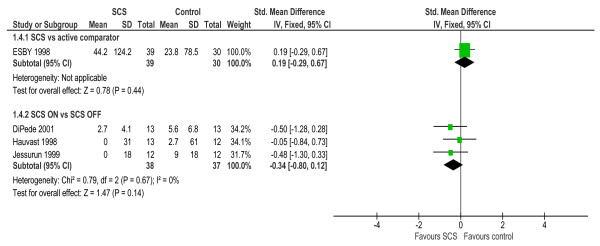
**Ischemic burden – between-group difference**.

Ischemic burden (i.e. frequency and magnitude of ST-depression over 24-hour or 48-hour ECG monitoring) was assessed in four studies [19,26-28]. In the two studies that reported the between group change in ischemic burden following SCS implantation, one reported a significant improvement at follow up while the other reported no change (Table 3). There was no significant difference in pooled ischemic burden at follow up between SCS and CABG (SMD: 0.19, 95% CI: -0.29 to 0.67, p = 0.44, see Figure 3) while there was a trend towards a lower ischemic burden with SCS compared to no SCS or SCS OFF (SMD: -0.34, 95% CI: -0.80 to 0.12, p = 0.12, Figure 3).

#### Nitrate drug consumption

**Figure 4:**
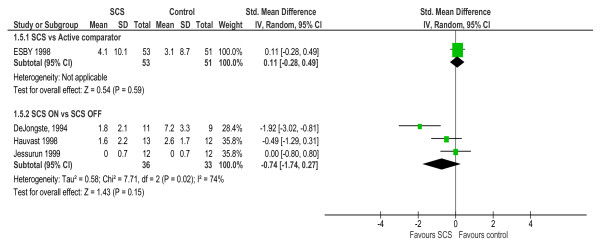
**Nitrate drug consumption – between-group difference**.

Five studies reported the consumption of shorting acting nitrates (GTN) [19,24,28,29]. All three studies reporting the between group change reported a significant reduction in GTN following SCS (Table 3). As shown in Figure 4 although the pooled analysis showed some evidence of lower level of GTN usage with SCS compared to no SCS/SCS OFF (SMD -0.74, 95% -1.74 to 0.27, *p *= 0 15, Test of heterogeneity; χ^2 ^= 7.7, *p *= 0.02, I^2 ^= 74%) this difference did not achieve statistical significance. There was no difference in nitrate consumption compared to CABG at 6-months or 2-years follow up (see Figure 4).

#### Functional class

Two studies reported the change in CCS class. The SPiRiT study found a lower CCS class at 3 and 12-months follow up in SCS treated patients compared to patients receiving PMR (3-months: *p *= 0.049; 12-months; *p *= 0.093) [23]. Eddicks et al reported a mean CCS of 1.6 in patients when receiving SCS (3–5.5 V 3 × 2 hours per day) compared to control treatment (0.1 V 24 hours per day) mean CCS of 3.1 (*p *= 0.002) [30].

#### Health- related quality of life

**Figure 5:**
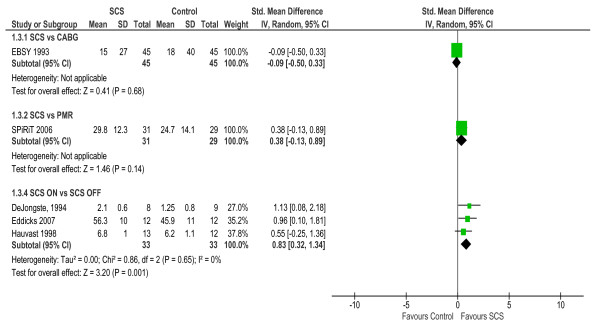
**Health related quality of life – between-group difference**. Based on NHP part 1 score for ESBY 1993; SF-36 physical health scale at 2-years for SPiRiT, 2006; ADL score for DeJongste 1994, EQ-5D VAS score for Eddicks 2008; LASA score for Hauvast 1998.

Five studies assessed health-related quality of life using validated instruments that included both generic (e.g. Short-Form 36, Nottingham Health Profile & EuroQoL (or EQ-5D)) and disease specific measures (Seattle Angina Questionnaire & Quality of Life Questionnaire – Angina Pectoris) [21,23,24,27,29]. The four studies that reported outcome at baseline and follow up, each found a significant improvement in quality of life (both generic and disease specific) with SCS (Table 3). Based on aggregate scores, pooled quality of life was superior for SCS compared to no SCS or SCS OFF (SMD: 0.83, 95% CI: 0.32 to 1.34, *p *= 0.001; Test of heterogeneity: χ^2 ^= 0.9, *p *= 0.65, I^2 ^= 0%). There was no significant difference in quality of life with SCS compared to either CABG or PMR (see Figure 5).

#### Adverse events SCS-related complications

Four studies reported data on SCS-related complications and adverse outcomes including cardiac events and treatment cross over (see Table 4) [19,23,25,27,29]. SCS-reported related complications included infections (1 out 104 patients, 1.0%) and lead migration or fracture (10 out of 128 patients, 7.8%). The risk of non-fatal events, fatal events and treatment cross-over appeared similar for SCS to that of PMR at 1 and 2-years follow up [23]. In the ESBY study, morbidity was comparable between the groups, however, total mortality was somewhat higher in the CABG group than with SCS (6 months follow up: 7/51 vs. 1/53, *p *= 0.02; 2 years follow up: 10/53 CABG 5/53, P = 0.16; 5 years follow up 16/51 vs. 13/53, *p *> 0.05) [19].

**Table 4:** Summary of safety outcomes – number of patient events

	**DeJongste 1994**	**Hauvast 1998**	**Eddicks 2007**	**ESBY 1998**	**SpiRiT 2006**
Follow up	13.8 months	6-weeks	4 weeks	2-years	1-year

**SCS-related complication**					

Infection	NR	0/13	NR	1/57	0/34
Lead migration/fracture	6/34	0/13	NR	3/57	1/34

**Mortality/morbidity**					

Non fatal MI	1/12	NR	NR	SCS 7/53 CABG 3/53, P = 0.18	SCS 4/34 PMR 1/34, P = 0.16
Total mortality	2/12	NR	NR	SCS 5/53 CABG 10/53, P = 0.16	SCS 4/34 PMR 2/34, P = 0.73
Cardiac mortality	2/12	NR	NR	SCS 3/53 CABG 5/53, P = 0.54	SCS 2/34 PMR 1/34, P = 0.52

**Other outcomes**					

Treatment cross over	NR	NR	NR	6-months SCS 3/53 CABG 3/51	SCS 1/34 PMR 0/33

CABG: coronary artery bypass graft; PMR: percutaneous myocardial laser revascularisation; NR: not reported

#### Healthcare utilisation and costs

Only one trial reported the use of healthcare resources and costs [18]. The ESBY trial reported healthcare utilisation and costs at 2-years follow up. The overall number of days of hospitalisation in patients receiving SCS (mean 5.0 days) was lower compared to those having undergone CABG (mean: 11.1 days, p < 0.0001). Taking into account the cost of the primary intervention, hospital days and follow up treatments and visits the average cost was 16,400 Euros per patient with SCS compared to 18,800 Euros per patient with CABG (p < 0.01).

## Discussion

To the author's knowledge this is the first published meta-analysis of SCS for the treatment of refractory angina.

We found seven RCT's of SCS recruiting a total of 270 patients. The inclusion criteria and patients characteristics of these trials were broadly similar i.e. NYHA class III and IV patients, predominantly male and post-myocardial infarction, experiencing angina in spite of optimal medical therapy with no foreseen benefit from CABG. Of these, two compared SCS to an active intervention (CABG or PMR) and the remainder compared SCS to a 'no stimulation' control. Meta-analysis showed that when compared to a no stimulation control, SCS significantly improved the exercise capacity and health-related quality of life of this patient population. The magnitude of outcome improvement with SCS appears to be similar to that of CABG and PMR, although the relevance of CABG as a treatment option for management of refractory angina is questionable [1,3]. A proportion of patients experienced SCS-related complications, in particular infections and lead migration or breakage. However, these complications are relatively minor and correctable. It has been suggested that SCS may mask angina symptoms and therefore increase the cardiac risk [9]. However, we found no increase in cardiac morbidity or mortality with SCS when compared to other treatments. This has also been reported in observational studies [9-12].

### Mechanism of action

The mechanism of action of SCS is not yet completely understood. A recent review of experimental studies provides a summary of putative mechanisms [21]. The benefits to the heart are not likely only to be due to an increase in blood flow. As recently shown, a redistribution of local blood flow may very well be responsible for the improvement in myocardial ischemia [9]. SCS is also associated with normalisation of the intrinsic cardiac nervous system [31].

### Limitations

The general lack of reporting of methods in the included RCTs made it difficult to assess their methodological quality and thereby judge their risk of bias and potential to overestimate the effect of SCS. One particular challenge of SCS evaluation is the difficulty in patient blinding as the therapy produces paraesthesia that must be elicited in the area of the pain if SCS is to be efficacious. In addition, the implantation procedure might in itself produce a placebo effect, but sham operations are ethically difficult to justify [32]. Thus a placebo effect of treatment cannot be completely excluded. However, it seems unlikely that a placebo effect alone would totally account for the clinical benefits from SCS seen in this review, including improvements in ischemic burden assessed by objective ambulatory ECG monitoring. By implanting a stimulator in both groups, a number of studies minimised the risk of an 'operation bias' [27].

Small trials are likely to have insufficient statistical power to detect potentially important clinical differences between treatments. In order to overcome this limitation, we pooled the results of ON-OFF SCS trials. A random effects meta-analysis approach was chosen to reflect both the clinical and methodological across trials. However, given the small number of trials we acknowledge that this statistical approach may have underestimated the between-trial variance resulting in the overall precision of treatment effect being over estimated and the resulting *p*-values from tests of treatment effects being too small.

Inevitably, any review can be subject to publication bias, i.e. studies with 'positive' results are more likely to be reported and published, while side effects and adverse events are more likely to be underreported [14]. Funnel plots (scatter plots of the treatment effects estimated from individual studies against each study's sample size) can be used to assess for the presence of publication bias i.e. the absence of negative trials [18]. Given the small number of RCTs it was difficult in this case to appropriately interpret the funnel plots. However there was some evidence of funnel plot asymmetry (not shown) in the most commonly reported outcomes in this review (i.e. exercise capacity, ischemic burden and nitrate consumption). Although such asymmetry can be indicative of publication bias (i.e. the absence of negative studies) it can also reflect poor methodological quality of smaller studies, true heterogeneity, size of effect differs according to study size (for example, due to differences in the intensity of interventions or differences in underlying risk between studies of different sizes) and chance [18].

### Clinical and future research implications

On balance, the finite evidence base of seven RCTs of moderate quality identified by this review supports the current 2007 ACC/AHA Grade IIb evidence classification and their Level B recommendation for SCS in refractory angina patients [13]. Given the relatively sparse evidence base for alternative treatments, these results would support the more widespread use of SCS in the management of refractory angina, particularly in the context of a clinical trial.

Additional high quality RCT and collected in appropriately selected patients is needed before SCS can be recommended for the routine treatment of refractory angina. In view of lack of consensus in the clinical community as to how to best manage refractory angina any future trial will need to carefully consider the choice of the comparator therapies. Such a trial will also need also to examine the question of value for money and therefore comprehensively assess healthcare resource utilisation, costs, and collect generic preference-based quality of life data such as the EQ-5D. The recently published 24-month analysis of the clinical and cost effectiveness of the SpiRiT trial provides a useful framework of the type of data analysis and outcomes needed in the future for SCS [33].

## Conclusion

SCS appears to be an effective and safe treatment option in the management of refractory angina patients and of similar efficacy and safety to PMR, a potential alternative treatment. Further high quality RCT and cost effectiveness evidence is needed before SCS can be accepted as a routine treatment for refractory angina.

## Abbreviations

ACC: American College of Cardiology; AHA: American Heart Association; CABG: coronary artery bypass surgery; CCS: Canadian Cardiovascular Society; NYHA: New York Heart Association; CI: confidence interval; ITT: intention to treat; NR: not reported; PMR: percutaneous myocardial laser revascularisation; SCS: spinal cord stimulation; RCT: randomised controlled trial; SMD: standardised mean difference

## Competing interests

Medtronic UK provided funding to the University of Birmingham and Peninsula Medical School to support the undertaking of this project including meetings of the international research team. None of the authors received personal remuneration from this funding. The planning of this study, interpretation of findings, writing and conclusions of manuscript were undertaken entirely independently of the company interests.

MDeJ and JdeV declare no competing. EHC Hospital of Morges (EB's employer) and RST have received financial reimbursement as consultants for Medtronic.

## Authors' contributions

RST, MdJ and JDeV conceived the study. RST analysed the data. All authors participated in the interpretation of results and drafting and approval of the manuscript.

## Appendix 1. Search strategy

Database: Ovid MEDLINE(R) <1950 to January Week 5 2008>

1 electric stimulation therapy.mp. or exp Electric Stimulation

Therapy/

2 exp Spine/

3 (spine or spinal or spines).mp.

4 or/2–3

5 1 and 4

6 scs.mp.

7 dorsal cord stimulat$.tw.

8 spinal cord stimulat$.tw.

9 neurostimulation.mp.

10 neuromodulation.mp.

11 or/5–10

12 angina pectoris.mp. or exp Angina Pectoris/

13 refractory angina.mp.

14 angina.mp.

15 or/12–14

16 11 and 15

17 randomized controlled trial.pt.

18 controlled clinical trial.pt.

19 randomized controlled trials.sh.

20 random allocation.sh.

21 double blind method.sh.

22 single-blind method.sh.

23 or/17–22

24 (animals not human).sh.

25 23 not 24

26 clinical trial.pt.

27 exp clinical trials/

28 (clin$ adj25 trial$).ti,ab.

29 ((singl$ or doubl$ or trebl$ or tripl$) adj25 (blind$ or mask$)).ti,ab.

30 placebos.sh.

31 placebo$.ti,ab.

32 random$.ti,ab.

33 research design.sh.

34 or/26–33

35 34 not 24

36 35 not 25

37 comparative study.sh.

38 exp evaluation studies/

39 follow up studies.sh.

40 prospective studies.sh.

41 (control$ or prospectiv$ or volunteer$).ti,ab.

42 or/37–41

43 42 not 24

44 43 not (25 or 36)

45 25 or 36 or 44

46 16 and 45

## Pre-publication history

The pre-publication history for this paper can be accessed here:


